# Combined detection and introgression of QTL in outbred populations

**DOI:** 10.1186/1297-9686-42-16

**Published:** 2010-06-03

**Authors:** M Hossein Yazdi, Anna K Sonesson, John A Woolliams, Theodorus HE Meuwissen

**Affiliations:** 1 Department of Animal and Aquacultural Sciences, Norwegian University of Life Sciences, Box 1432 Ås, Norway; 2 Nofima Marine AS, P.O. Box 5010, 1432 Ås, Norway; 3 The Roslin Institute, Royal (Dick) School of Veterinary Studies, University of Edinburgh, Roslin, Midlothian, EH25 9PS, UK

## Abstract

**Background:**

Detecting a QTL is only the first step in genetic improvement programs. When a QTL with desirable characteristics is found, e.g. in a wild or unimproved population, it may be interesting to introgress the detected QTL into the commercial population. One approach to shorten the time needed for introgression is to combine both QTL identification and introgression, into a single step. This combines the strengths of fine mapping and backcrossing and paves the way for introgression of desirable but unknown QTL into recipient animal and plant lines.

**Methods:**

The method consisting in combining QTL mapping and gene introgression has been extended from inbred to outbred populations in which QTL allele frequencies vary both in recipient and donor lines in different scenarios and for which polygenic effects are included in order to model background genes. The effectiveness of the combined QTL detection and introgression procedure was evaluated by simulation through four backcross generations.

**Results:**

The allele substitution effect is underestimated when the favourable QTL allele is not fixed in the donor line. This underestimation is proportional to the frequency differences of the favourable QTL allele between the lines. In most scenarios, the estimates of the QTL location are unbiased and accurate. The retained donor chromosome segment and linkage drag are similar to expected values from other published studies.

**Conclusions:**

In general, our results show that it is possible to combine QTL detection and introgression even in outbred species. Separating QTL mapping and introgression processes is often thought to be longer and more costly. However, using a combined process saves at least one generation. With respect to the linkage drag and obligatory drag, the results of the combined detection and introgression scheme are very similar to those of traditional introgression schemes.

## Background

In QTL mapping designs such as those using F2 or backcross animals, the power to detect QTL is based on the assumptions that all genes affecting the trait of interest are biallelic with alternative alleles fixed in each parental inbred line and that there is no genetic variation within the line. In some plant species and laboratory animals, highly inbred lines are available that may fulfil this condition, but many important species are outbreeders: such as livestock (e.g., [1]), trees (e.g., [2]), fish (e.g., [3]), as well as most wild species (e.g., [4]).

However, detecting a QTL is only the first step in genetic improvement programs. When a QTL with desirable characteristics is detected e.g. in wild or unimproved populations, it may be desirable to introgress it into the commercial population. One approach to shorten the time needed for introgression is to combine both, QTL identification and introgression, into a single step. This combines the strengths of fine mapping and backcrossing and paves the way for introgression of desirable but unknown QTL into recipient animal and plant lines [5]. Combining QTL identification and introgression corresponds to a continuous backcrossing scheme, where the information of the backcross generations is used to identify and map the QTL. Whilst previous work has shown the benefit of combining QTL mapping and gene introgression [5], the method applied only to inbred lines has a major limitation.

The objective of this study was to extend the approach of Yazdi et al. [5]. We will focus primarily upon instances where the recipient line does not carry the favourable QTL allele, since otherwise a marker assisted selection scheme can be used (e.g. [6]). The effectiveness of this method was investigated through computer simulation considering two outbred lines, in which QTL alleles were segregating and polygenic effects were included.

## Methods

### Genome structure

In this study, we simulated individuals with a genome consisting of one 100 cM chromosome and including a polygenic effect, i.e. assuming many genes each with a small effect. The polygenic effect was assumed to be independent from the QTL effect within lines but in linkage disequilibrium with the QTL effect between lines. The chromosome carried a single QTL with a major effect on the trait of interest located at 84.5 cM from the beginning of the chromosome, and it included 101 anonymous markers, positioned at the ends and at 1 cM intervals along the chromosome. The QTL was positioned so that it was neither around the chromosome's centre or ends nor located at a marker position. Positions at the chromosome's centre and ends were avoided respectively because QTL mapping methods can show a bias towards the centre of the considered segment [7] and because an end location would result in truncated likelihood peaks which are unsatisfactory for assessing the procedures proposed. Each locus, either QTL or marker, was assumed to be biallelic with additive gene effects for the QTL and no effects for the markers.

Two founder outbred lines were considered: a donor line containing a favourable QTL allele with a high frequency and a recipient line considered to be highly desirable for other traits. Throughout this report, subscripts'd' and 'r' represent donor and recipient lines, respectively. For the donor line, marker loci and QTL were both assumed to be biallelic with alleles, M or m for markers, Q or q for QTL, where M and Q are the major alleles, and where Q is the favourable allele at the QTL locus. The allelic frequency in the donor line *p*(*Q*_*d*_) was varied as described later. When markers and QTL segregated within lines, they were considered to be in pair-wise linkage equilibrium, which is a conservative assumption since there is no population-wide linkage disequilibrium (LD) contributing information. Within the recipient line the QTL was considered to be fixed for the minor allele. In another set of scenarios, the recipient line was considered complementary to the donor line for the frequencies of the QTL alleles; for example, if the major allele had a frequency of 0.9 in the donor line, its frequency was *p*(*Q*_*r*_) = 1 - *p*(*Q*_*d*_) = 0.1 in the recipient line (see Table 1). Therefore, when, *p*(*Q*_*d*_) = 0.5, there is no difference in QTL allele frequencies between lines.

**Table 1:** QTL allele frequencies^a ^and genotypes of individuals in the base outbred lines and their first backcross (BC1) generation

Allele in outbred lines		BC1 genotype			
*P*(*Q*_*d*_)	*P*(*Q*_*r*_)	*P*(*Q*_*d*_*Q*_*r*_)	*P*(*Q*_*d*_*q*_*r*_)^b^	*P*(*q*_*d*_*Q*_*r*_)	*P*(*q*_*d*_*q*_*r*_)
1.0	0.0	0.00	0.50	0.00	0.00
0.90	0.0	0.00	0.45	0.00	0.05
0.75	0.0	0.00	0.38	0.00	0.12
0.50	0.0	0.00	0.25	0.00	0.25
0.90	0.10	0.05	0.41	0.01	0.05
0.75	0.25	0.09	0.28	0.03	0.09
0.50	0.50	0.13	0.12	0.12	0.13

^a ^Subscripts of ***d ***and ***r ***represent donor and recipient outbred lines, respectively

^b ^Individuals with these genotypes in BC1 generation are informative

The remaining genotype not shown is *q*_*r*_*q*_*r*_, which accounts for all remaining frequencies in backcross generations, e.g. when *P*(*Q*_*d*_) = 1.0 and *P*(*Q_r_*) = 0.0, *P*(*q_r_q_r_*) = 1.0 - 0.5 = 0.5

### Base populations, selection and mating

The outbred recipient and donor lines were simulated using Monte Carlo simulation. In the base population, two QTL alleles were randomly sampled for each animal. In addition to the effect of the major QTL, the recipient and donor lines were assumed to have developed over generations from a common base generation with a genetic variance . The genetic variance within both lines () was derived from the common base population (see Appendix). The difference between the two lines for the trait of interest was considered to be 1*σ*_*w *_unit in favour of the donor, and was assumed to be due to genetic drift. This genetic difference ignored the QTL and the markers which were assumed to be mutations having occurred later.

Introgression was carried out by crossing the outbred lines to produce an F1 generation, and then by recurrent backcrossing of the selected individuals from the crossbred population to the recipient line, to produce generations BC_1_, BC_2_, BC_3 _and BC_4_. In this study, BC_4 _was the last backcross generation considered. All generations were discrete and consisted of N individuals. In this population structure, recurrent parents come from the recipient line, and non-recurrent parents are the selected F1, BC_1_, BC_2 _and BC_3 _individuals.

In each generation, selection was based on the probability that the candidate is heterozygous for the QTL, conditional on the marker information. Individuals were selected if the probability of being heterozygous exceeded a predetermined threshold value of 0.95. As a consequence, a variable number of candidates was selected and given an opportunity to breed. The calculation of this selection criterion will be described in the QTL mapping section.

Mating took place randomly to reproduce *N *offspring (1/2 *N *males, 1/2 *N *females). For each offspring, a sire and dam were chosen at random from among the selected ones. In each generation, crossing-over events were generated according to Haldane's [8] mapping function. A gamete passing from a parent to an offspring had an equal chance of carrying the paternal or maternal chromosome sequence and if a recombination occurred the reading sequence switched to the alternative parental chromosome. The polygenic value of the offspring was calculated as:

Where a_offspring _is the Mendelian sampling term for the offspring and was randomly sampled from a Normal distribution of mean 0 and variance , where t denotes the generation. Due to crossing of lines, the magnitude of  will vary and the approach to calculate  is given in the Appendix. The values obtained are:

A phenotypic record for each individual was simulated based on the following model:

Where *y*_*i *_is the phenotypic value of the i^th ^individual (*i *= 1 ... *N*), *μ *is the population mean, g is the mean difference between donor and recipient lines, *c*_*i *_is the donor line contribution to individual i for the polygenic effect which decreases from 1/2 to 0 from F_1 _onwards, *a*_*i *_is the animal's polygenic effect obtained as described above, *b*_*i *_is an indicator variable which takes the value 1 when carrying the favourable QTL allele and otherwise is 0, *α *is the allele substitution effect of the favourable QTL allele, and *e*_*i *_is a random normal variable with mean 0.0 and variance . The QTL effect was assumed additive, but this assumption can be relaxed (see Yazdi et al., 2008 [5]).

### QTL mapping

The single interval mapping regression model [9] was applied for QTL mapping. In this model, one marker interval at a time was used to construct a putative QTL likelihood at the midpoint location of the interval. For each generation in the backcross program, using marker information for individual i and interval j, denoted by *M*_*ij*_, with the phenotypic value y_i _of the recorded trait, a mixed model for a putative QTL at the interval's midpoint *x*_*j *_was fitted. From generation BC1 onward, all the accumulated phenotypes from the previous generations were used in the model to estimate the QTL locations and effects. Therefore the following model was used for each interval in each generation:(1)

Where ***y ***is a vector of observations in the backcross generation t for t = 1 ... 4, *μ *is the overall mean; γ is a vector of generation effects for average genetic merit, *α *is fixed effect of favourable QTL allele;  is a vector of animal polygenic values,  is a vector of residual effects; ***A ***is the matrix of additive genetic relationships among animals assuming that the recipient and donor lines were unrelated; ***1 ***is a vector with each element 1, ***X***_1 _is a design matrix for effect of generation, ***X***_2 _is a vector of probabilities of the QTL genotypes π(*Qq*|*M*_*ij*_) conditional on marker genotypes and position of the flanking markers, described in more detail below. The ***Z ***is an incidence matrix that assigns the animal's effects to the vector of observations.

The probability of the QTL genotype π(*Qq*|*M*_*ij*_) was calculated based on the marker genotype of the individual and its non-recurrent (backcross) parent at flanking markers in each interval, assuming that marker phases are known. Calculation of π(*Qq*|*M*_*ij*_) was based on the recombination fractions *θ*_1 _and *θ*_2 _between the QTL and the heterozygous flanking markers of the non-recurrent parent [10]. If a marker locus of the non-recurrent parent was non-informative then the interval was expanded until the next heterozygous marker locus [5]. Heterozygous markers of non-recurrent parents were assumed informative, through the combination of known marker phases and closely linked flanking markers, so that the recurrent or non-recurrent grand-parent of both alleles could be inferred. However, the value of π(*Qq*|*M*_*ij*_) was not conditioned on the phenotypes in the population, so once calculated, the π(*Qq*|*M*_*ij*_) remains constant over generations for each QTL position. The markers information was used to trace the line of origin, and hence the QTL genotype was based on this information. As developed in the discussion, it is possible to improve the calculation of the probability of heterozygous parents by including phenotypic information; hence relying only on identification of the original line is a conservative assumption.

Parameters were estimated using the average information algorithm for restricted maximum likelihood (AI-REML) included in the DMU-package of Madsen and Jensen [11]. The convergence criterion was chosen so that the norm of the update vector for the (co)variance components was less than 10-^8^. The interval with the highest maximized likelihood values was taken as the estimated location of the QTL, and the estimate of effect  for this interval was taken as the estimate of the QTL allele substitution effect.

The selection criterion for selecting carrier (*Qq*) parents was the probability that the individual carries the favourable donor allele at the estimated QTL locations given the marker information. Individuals that were heterozygous at the estimated QTL location with a probability *π*(*Qq*|*M*_*iτ*_) ≥ 0.95 were selected, where *τ *is the estimated location of the QTL with the highest probability across all intervals. Hence there was a possibility that some non-carrier parents were selected erroneously. However, no attempt was made to remove these errors.

### Parameters and simulations

In this study, two different values of N (500 or 1000), four frequencies of the favourable QTL allele in the donor line (1.0, 0.90, 0.75 or 0.50), and three heritability values (*h*^2 ^= 0.50, 0.31 or 0.17) were considered. For one set of scenarios with all four values of, *p*(*Q*_*d*_), the recipient line was assumed to be fixed for all m and q alleles. In these scenarios, *p*(*M*_*d *_= *p*(*Q*_*d*_), although as stated above, marker loci and QTL were in pair-wise linkage equilibrium. In another set, allele Q was considered as segregating in the recipient line, with *p*(*Q*_*r*_) = 1 - *p*(*Q*_*d*_) with *p*(*Q*_*d*_) = 0.90 or 0.75. Marker loci in the recipient and donor lines were segregating with *p*(*M*_*r*_) = *p*(*M*_*d*_) = *p*(*Q*_*d*_) as described above.

Three different sizes of the QTL effect were considered: α = 2.23, 1.48, and 1.02, where α is the allele substitution effect of the QTL. If the allele frequency in the donor line was 1, this generated a genetic variance due to the QTL of 1.24, 0.548, and 0.260, respectively, and the polygenic variance was assumed three times bigger than the QTL variance, i.e. 3.713, 1.65 and 0.782. If heritability is defined as the sum of the QTL and polygenic variances divided by this same sum plus the environmental variance, then the heritability values are 0.50, 0.31 and 0.17, respectively. Although we will differentiate between the schemes by referring to these heritability values, it should be noted that the actual heritability in any one generation may differ from these heritability values due to (i) differences in allele frequencies at the QTL alleles and (ii) changes in the Mendelian sampling variance as described in the Appendix. Simulations were replicated 100 times.

For each replicate, the efficiency of selection, the donor genome contribution and the linkage and obligatory drags at BC1 and BC4 were calculated from direct examination of the marker sequence along the genome of individuals with respect to the estimated QTL location [5]. The efficiency of selection is calculated as the ratio of the number of selected individuals that are heterozygous for the actual QTL to the total number of selected individuals. The donor genome contribution is the fraction of the backcross genome that derives from the donor genome. The linkage drag is the average length of the intact segment of the donor genome flanking the QTL, whereas the obligatory drag is the minimum segment length of the donor genome to the left and to the right of the QTL across the whole population, which represents the part of the donor genome that cannot be removed from an intercross formed from the final generation.

## Results

Frequencies of QTL alleles and genotypes of individuals in the base outbred lines and their backcross (BC) generations are presented in Table 1 for all studied cases. Since the frequencies of genetic markers were the same as those of the QTL in the donor line, they are not shown. Heterozygous individuals for which the favourable QTL allele originated from the donor line, *Q*_*d*_*q*_*r*_, are informative in the sense that they contribute to the accuracy of the QTL mapping as formulated. As the frequency of the favourable allele in the donor line decreases from 1, the proportion of individuals with the informative *Q*_*d*_*q*_*r *_genotype is reduced (column 4 in Table 1).

### Recipient's marker loci and QTL fixed for the donor's minor allele

In Tables 2 and 3, results are presented for 12 different scenarios, where Q is segregating at one of four frequencies in the donor line (*P*(*Q*_*d*_) = 1.0, 0.90, 0.75 and 0.50) and is not segregating (*P*(*Q*_*r*_) = 0.0) in the recipient line and where three heritability values (*h*^2 ^= 0.50, 0.31 or 0.17) are considered. The estimates of the QTL allele substitution effect () were comparable to the true values when the favourable QTL allele was fixed, *P*(*Q*_*d*_) = 1.0 in the donor line (Table 2). However, estimates of QTL allele substitution effects were underestimated as the frequency of favourable QTL allele decreased from 1 in the donor line. For example, when *P*(*Q*_*d*_) = 5.0, only 50% of the F1 individuals carried the favourable QTL allele from the donor line when it was heterozygous for linked markers because of the linkage equilibrium assumed in the simulated data. Based on the selection criteria, only 50% of the selected parents were truly heterozygous for the QTL while the remaining were falsely assumed to be heterozygous, and consequently the estimate of  was about 50% of the true values. In general, when the frequency of the favourable QTL allele in the donor line decreases, which corresponds to a decreasing effect of the donor chromosome segment, the estimate of  is also reduced. There was no evidence of an association between this bias and the heritability.

**Table 2:** Estimates of QTL allele substitution effect (), location and efficiency of selection in BC1 and BC4 when frequency of favourable QTL allele in donor line varied and N = 1000 (*se *are in *italic *font).

*α*^*b*^			**Location **^**c**^		Efficiency of selection	
	BC1	BC4	BC1	BC4	BC1	BC4
*p*(*Q*_*d*_) = 1.00						
2.23	2.23 *± 0.02*	2.21 *± 0.01*	84.8 ± *0.1*	85.0 ± *0.0*	1.00 ± *0.00*	0.99 ± *0.00*
1.48	1.49 *± 0.02*	1.47 *± 0.01*	85.1 ± *0.2*	85.0 ± *0.1*	0.99 ± *0.00*	0.99 ± *0.00*
1.02	0.99 *± 0.02*	0.96 *± 0.02*	83.0 ± *1.5*	82.8 ± *1.5*	0.99 ± *0.00*	0.98 ± *0.00*
*p*(*Q*_*d*_) = 0.90						
2.23	2.04 *± 0.02*	2.00 *± 0.01*	85.0 ± *0.1*	85.1 ± *0.1*	0.90 ± *0.00*	0.90 ± *0.00*
1.48	1.35 *± 0.02*	1.32 *± 0.01*	84.8 ± *0.2*	84.9 ± *0.1*	0.89 ± *0.00*	0.89 ± *0.00*
1.02	0.95 *± 0.02*	0.92 *± 0.01*	85.1 ± *0.4*	85.0 ± *0.1*	0.89 ± *0.00*	0.88 ± *0.00*
*p*(*Q*_*d*_) = 0.75						
2.23	1.67 *± 0.02*	1.64 *± 0.01*	84.9 ± *0.3*	85.0 ± *0.1*	0.75 ± *0.00*	0.74 ± *0.00*
1.48	1.12 *± 0.02*	1.09 *± 0.01*	84.9 ± *0.3*	85.1 ± *0.1*	0.74 ± *0.00*	0.73 ± *0.00*
1.02	0.80 *± 0.02*	0.76 *± 0.01*	84.1 ± *0.7*	84.9 ± *0.2*	0.72 ± *0.01*	0.72 ± *0.00*
*p*(*Q*_*d*_) = 0.50						
2.23	1.14 *± 0.02*	1.09 *± 0.01*	84.3 ± *0.5*	85.0 ± *0.1*	0.50 ± *0.00*	0.49 ± *0.00*
1.48	0.76 *± 0.02*	0.72 *± 0.01*	82.3 ± *0.2*	85.0 ± *0.2*	0.48 ± *0.01*	0.48 ± *0.01*
1.02	0.57 *± 0.02*	0.51 *± 0.01*	81.2 ± *1.4*	85.2 ± *0.4*	0.48 ± *0.01*	0.47 ± *0.01*

^a ^Marker frequencies and QTL frequencies were identical in the donor line

^b ^True QTL allele substitution effect; allele substitution effects of 2.23, 1.48 and 1.02 correspond to heritability values of 0.50, 0.31 and 0.17, respectively

^c ^True QTL location corresponds to interval 85

The estimate for the QTL location in most scenarios was close to the true interval (85) in both BC1 and BC4 generations (Table 2). When the frequency of the favourable QTL allele in the donor line was 0.5 with the lowest heritability values, the estimates of the QTL location were biased (i.e. at *α *= 1.02 at and BC1). The direction of the bias for QTL location is towards the centre of the chromosome as is expected when the QTL location is not estimated accurately [7]. The standard error of the QTL location increased slightly as the frequency of the favourable QTL allele decreased in the donor line together with decreasing heritability. However, the range of location estimates depends on the frequency of the favourable QTL in the donor line. For instance, when *P*(*Q*_*d*_) = 1.0 location estimates ranged between intervals 84 and 86, while when *P*(*Q*_*d*_) = 0.5 they ranged between intervals 62 and 98.

The efficiency of selection in BC1 and BC4 generations was lower if the frequency of the favourable QTL allele in the donor line was reduced (Table 2), which is directly linked to the frequency of informative individuals in Table 1. This decreasing efficiency of selection partially explains the underestimates of , because the method estimated the average effects of the QTL allele coming from the donor line, which is underestimated if the donor line has a low frequency of the favourable QTL allele. Comparing BC1 and BC4, efficiencies of selection across generations were very similar. In general, the accuracy of the estimates of efficiency of selection was high and the replication error was very low. It should be noted that the efficiency of selection also reflects the reduced number of selected animals.

The estimated polygenic variances (Table 3) were overestimated when the QTL effect was underestimated, i.e. it picked up the generic variance that was not explained by fitting the QTL. The genome contribution of the donor line after four backcross generations ranged from 41.5 to 44.2 cM across the different QTL allele frequencies in the donor line and the different QTL allele substitution effects. Since the genome was 100 cM long, all values in cM can be considered as proportional. It should be noted that there was no background selection in this study. Likewise linkage drag was also reasonably consistent across scenarios, ranging from 36.2 to 38.7 cM across all scenarios. Although there was no significant difference between linkage drags across the different heritabilities and frequencies of the favourable QTL allele in the donor line, there was a trend for a lower linkage drag when the frequency of the favourable QTL allele in the donor was reduced. The obligatory drag ranged from 2.1 to 2.3 cM with a slight increasing trend in conjunction with a lower frequency of the favourable allele in the donor line. The standard error of the obligatory drag was very low and similar across the different frequencies of the favourable QTL allele in the donor line and the different QTL allele substitution effects. The number of selected individuals was under 50% but usually close to this value, which is the upper bound of our expectation because only 50% of the animals are heterozygous. As the frequency of the favourable allele in the donor line decreases, the number of non-informative individuals increases (Table 1). The estimated residual variance was close to the true value () and did not deviate significantly (*p *≤ 0.05) from the true value in all scenarios.

**Table 3:** Estimates of residual variance, donor genome contributions and efficiency of selection in BC4 when N = 1000 (*se *are in *italic *font).

*h*^2^		Donor genome(cM)	Linkage drag (cM)	Obligate drag (cM)	Number of selected individuals
*p*(*Q*_*d*_) = 1.00					
0.50	3.74 *± 0.03*	41.50 *± 0.13*	36.17 *± 0.13*	2.06 ± *0.02*	491 *± 1*
0.31	1.68 *± 0.02*	41.80 *± 0.15*	36.56 *± 0.14*	2.09 ± *0.03*	492 *± 1*
0.17	0.76 *± 0.02*	43.68 *± 0.77*	38.46 *± 0.76*	2.09 ± *0.03*	480 *± 7*
*p*(*Q*_*d*_) = 0.90					
0.50	3.97 *± 0.03*	41.68 *± 0.20*	36.28 *± 0.17*	2.06 ± *0.02*	492 *± 1*
0.31	1.79 *± 0.02*	41.78 *± 0.16*	36.37 *± 0.15*	2.10 ± *0.03*	490 *± 1*
0.17	0.86 *± 0.02*	42.00 *± 0.17*	36.86 *± 0.16*	2.09 ± *0.03*	490 *± 1*
*p*(*Q*_*d*_) = 0.75					
0.50	4.21 *± 0.03*	41.57 *± 0.18*	36.32 *± 0.17*	2.18 ± *0.04*	491 *± 1*
0.31	1.89 *± 0.02*	42.28 *± 0.19*	37.06 *± 0.17*	2.08 ± *0.03*	489 *± 1*
0.17	0.91 *± 0.02*	42.69 *± 0.20*	37.30 *± 0.18*	2.09 ± *0.03*	490 *± 1*
*p*(*Q*_*d*_) = 0.50					
0.50	4.26 *± 0.04*	42.25 *± 0.26*	36.96 *± 0.23*	2.17 ± *0.04*	490 *± 1*
0.31	1.92 *± 0.02*	42.83 *± 0.30*	38.73 *± 0.34*	2.29 ± *0.06*	484 *± 3*
0.17	0.93 *± 0.02*	44.22 *± 0.41*	38.73 *± 0.34*	2.29 ± *0.06*	484 *± 3*

^a ^Marker frequencies and QTL frequencies were identical in the donor line

^b ^True polygenic variance corresponding to heritability values *h*^2 ^= 0.50, 0.31, 0.17 were equal to 3.71, 1.65 and 0.78, respectively

Results for the 12 different scenarios (four values of *p*(*Q*_*d*_) for each of three *α *values), when N = 500 correspond to those in Tables 2 and 3, are not shown since there was a very similar pattern of estimation properties. Decreasing N resulted in greater underestimation of parameters when *p*(*Q*_*d*_) decreased. The magnitudes of donor genome contributions and linkage drags for N = 500 were greater than for N = 1000 due to the lower number of recombinations occurring.

**Figure 1:**
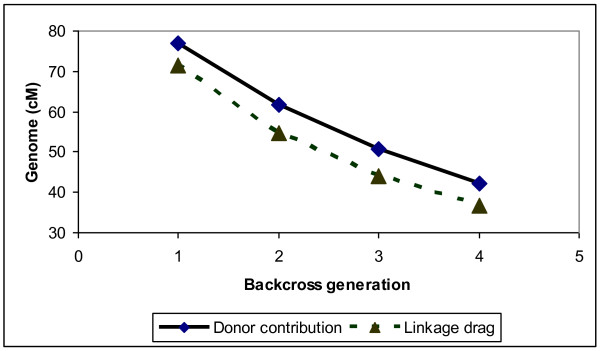
**Trend of donor genome contribution across different backcross generations**.

The size of the total donor genome and linkage drag that remained over the generations when the frequency of the favourable QTL allele in the donor line was 0.9, *α *= 1.48 and N = 1000 is illustrated in Figure 1. The total donor genome was ~77 cM long in BC_1 _and decreased to ~42 cM in BC_4_. The linkage drag also decreased from ~71 in BC_1 _to ~37 cM in BC_4_. Hence, as expected, the trend decreased over generations. Results of these parameters in the other scenarios were similar.

### Recipient's marker loci and QTL frequencies complementary to the donor's frequencies

In order to investigate the effects of variable frequencies of the favourable QTL allele in the recipient line, scenarios in which Q is segregating in the recipient line are presented in Table 4. Results are presented only for two different frequencies (0.10 and 0.25) of the favourable QTL allele in the recipient line and three heritability values and two population sizes. Here *P*(*Q*_*r*_) = 1.0 - *P*(*Q*_*d*_) can be compared to Table 2 where *P*(*Q*_*r*_) was 0 with the same *P*(*Q*_*d*_). The degree of underestimation of  was more severe. This was associated with greater bias in the estimate of the QTL location, which was more towards the centre of the chromosome as compared to the results for BC1 in Table 2. This was in part due to the identification of Q homozygotes with genotype *Q*_*d*_*Q*_*r *_as carriers for which the markers had no true information on position. The efficiency of selection is comparable to the results in Table 2.

**Table 4:** Estimates of QTL allele substitution effect (), location and efficiency of selection in BC1 and BC4 when N = 1000 (*se *are in *italic *font).

*α*^*b*^			**Location**^**c**^		Efficiency of selection	
	BC1	BC4	BC1	BC4	BC1	BC4
*p*(*Q*_*d*_) = 0.90, *p*(*Q*_*r*_) = 0.10 and *population *= 1000						
2.23	1.81 *± 0.02*	1.77 *± 0.01*	85.1 ± *0.2*	85.1 ± *0.0*	0.91 ± *0.00*	0.90 ± *0.00*
1.48	1.16 *± 0.02*	1.18 *± 0.01*	85.2 ± *0.3*	84.8 ± *0.1*	0.90 ± *0.00*	0.90 ± *0.00*
1.02	0.84 *± 0.01*	0.81 *± 0.01*	85.5 ± *0.4*	85.3 ± *0.1*	0.89 ± *0.00*	0.89 ± *0.00*
*p*(*Q*_*d*_) = 0.75, *p*(*Q*_*r*_) = 0.25 and *population *= 1000						
2.23	1.08 *± 0.03*	1.05 *± 0.02*	81.9 ± *1.7*	85.5 ± *0.3*	0.80 ± *0.00*	0.80 ± *0.00*
1.48	0.78 *± 0.02*	0.73 *± 0.01*	83.2 ± *1.2*	84.9 ± *0.3*	0.81 ± *0.00*	0.80 ± *0.00*
1.02	0.54 *± 0.02*	0.51 *± 0.01*	78.8 ± *1.6*	85.0 ± *0.3*	0.79 ± *0.00*	0.78 ± *0.00*
*p*(*Q*_*d*_) = 0.90, *p*(*Q*_*r*_) = 0.10 and *population *= 500						
2.23	1.80 *± 0.03*	1.49 *± 0.02*	84.9 ± *0.4*	84.8 ± *0.1*	0.90 ± *0.00*	0.89 ± *0.00*
1.48	1.25 *± 0.02*	1.02 *± 0.02*	83.4 ± *0.9*	84.8 ± *0.2*	0.89 ± *0.00*	0.87 ± *0.01*
1.02	0.83 *± 0.03*	0.69 *± 0.01*	80.5 ± *1.5*	85.0 ± *0.4*	0.87 ± *0.01*	0.85 ± *0.01*
*p*(*Q*_*d*_) = 0.75, *p*(*Q*_*r*_) = 0.25 and *population *= 500						
2.23	1.19 *± 0.03*	0.92 *± 0.02*	82.5 ± *1.2*	85.7 ± *0.3*	0.79 ± *0.01*	0.79 ± *0.01*
1.48	0.78 *± 0.03*	0.58 *± 0.01*	75.9 ± *2.2*	82.9 ± *0.7*	0.78 ± *0.01*	0.75 ± *0.00*
1.02	0.54 *± 0.03*	0.42 *± 0.01*	68.2 ± *2.7*	79.8 ± *1.1*	0.76 ± *0.01*	0.72 ± *0.01*

^a ^Marker frequencies and QTL frequencies are identical in the donor line

^b ^True QTL allele substitution effect; allele substitution effects of 2.23, 1.48 and 1.02 correspond to heritability values of 0.50, 0.31 and 0.17, respectively

^c ^True QTL location corresponds to interval 85

## Discussion

The QTL mapping and gene introgression approach is extended here to outbred populations, where it is assumed that the QTL allele frequencies may vary in both recipient and donor lines and where the polygenic effects are included in order to model the background genes. The process was qualitatively successful in detecting the QTL and integrating it progressively into a recipient line over several generations of backcrossing, although quantitatively the process resulted in underestimation of the allelic substitution effect (), unless the favourable QTL allele in the donor line was nearly fixed, i.e. *p*(*Q*_*d*_) and correspondingly absent in the recipient line.

Analysis of the results shown in Tables 2 and 4 indicates that this underestimation is proportional to the difference in allelic frequency *Q*_*d*_-*Q*_*r*_. For instance, in Table 2 when the frequency of the favourable QTL allele in the donor line is 0.75 and the recipient line has no Q alleles, the estimates of  are about 75% of the true values. In Table 4, when the frequency of the Q is 0.25 in the recipient line, the estimates of  are about 50% of the true values. This underestimation is in agreement with previous reports [12] on the detection and estimation of QTL in outbred lines. The estimate of  reflects the difference in genetic value between the introgressed chromosome segment of the donor line and that of the recipient line. Hence,  gives an unbiased estimate of the value of the introgressed donor segment, rather than the QTL, and this may be smaller than the QTL effect if the donor segment does not always carry the positive QTL allele or if the recipient segment already carries the positive QTL allele. This arises as a result of the mapping method which is concerned solely with the identification of the line of origin from the marker alleles.

In these models, complementarity of allelic frequencies was assumed, thus although a conservative assumption of linkage equilibrium was made within each line, LD between the QTL and markers would still occur in the F1 due to a difference in allele frequency between the lines, but would decline to 0 when the frequencies approach 0.5 within each line. However, there are two reasons to assume that the combined detection and introgression procedure could be used mainly when there is substantial LD in the F1 cross. Firstly it may be assumed that the marker density of maps might be sufficient to generate haplotypes predictive of the line's origin not only in the immediate vicinity of the marker locus, but also in the region spanning the marker loci. Secondly it might be expected that the more valuable recipient line would have been screened to identify QTL for the trait of interest that may have been segregating within it, before beginning the costly process of introgression, thus it is possible that the frequency of the donor line QTL is low only within the recipient line. Taken together, these arguments suggest that the simulation's assumptions of strong LD between QTL and markers might be a likely outcome in application.

When QTL are mapped in outbred populations, it is important to account for background genes, which are modelled as polygenic effects here, because the background genes may cause spurious associations between the markers and the trait (see Meuwissen and Goddard [13], for a review). Including a polygenic effect in the model, reduces spurious associations substantially, but does not guarantee that they do not occur. Therefore, here and in any other application of QTL mapping in outbred populations, one needs to remain aware that spurious associations may occur. However, the results suggest that spurious associations are less of a problem in the combined QTL detection and introgression schemes than in the standard QTL mapping in outbred populations schemes, because the LD generated by crossbreeding will probably overwhelm spurious associations [14-16].

In this study, the methodology used for QTL detection was very straightforward and conservative in the way the information was used, but it could be made more sophisticated. For example, lack of fixation at the QTL locus within either recipient or donor line could be included within the model [17]. The latter requires that the probability of the QTL genotype being *Q*_*d*_*Q*_*r *_is estimated not only from the marker data but also from the phenotypes. Conditional on the marker genotype, the data analysis then becomes a question of fitting a mixture model (one component distribution for each possible QTL genotype). This is a complicated model, especially since the polygenic effects need to be fitted simultaneously, but MCMC methods may be able to fit such a model. Furthermore, within-population LD between markers and QTL may be used to improve the estimates of QTL genotype probabilities [18]. If it is assumed that the donor and recipient populations are derived from a common ancestral population, across-population LD may be used to further improve the mapping precision. Therefore, although the result showed that mapping precision was quite good, even without using these additional sources of information, the use of more sophisticated methods may remove the biases observed in this study when estimating QTL effect and location.

The risks of carrying out such a combined QTL detection and introgression scheme will lie in false positive QTL and in location errors. The first risk may be controlled by setting high significance thresholds before accepting the presence of a QTL; given the cost of the process it seems sensible to set stringent thresholds. Concerning the second risk, inaccurate localization problems may be addressed by using quite wide confidence intervals for the QTL, i.e. introgressing a chromosome region most certainly carrying the QTL, although this will increase the linkage drag and the obligate drag. Another localization problem that may arise is the localization of a ghost QTL [9,19], i.e. a QTL peak that occurs in between two real QTL as the result of the joint effect of the two linked QTL. Improving the mapping precision, e.g. by including LD information, may reveal that there are actually two QTL underlying the original QTL signal.

In practice, there may still be a problem resulting from spurious associations arising from, say, LD over long distances leading to erroneous localization of the QTL. Such problems are more likely to occur with populations with a historically low effective size. Therefore, combined QTL mapping and introgression might be suitable for introgression of genes from wild ancestors in, say, sylvicultural or aquacultural settings. In agricultural species, a lower *N*_*e *_may demand more care.

Nevertheless, our results show that nothing prevents combining detection and introgression even in outbred species. As stated in Yazdi *et al. *[5] such a process is often thought of as two steps thus making it longer and more costly. However, using the combined process, it is possible to save at least one generation. As discussed by Yazdi *et al. *[5], with respect to the linkage drag and obligatory drag, the combined detection and introgression scheme and the traditional introgression schemes give very similar results.

## Appendix

### Appendix A - Approach to calculate variance within lines

It is assumed that recipient and donor lines have drifted independently from a base population with variance . By the time of introgression, variance within each line is , and the observed squared difference in means (*μ*_*R *_- *μ*_*D*_)^2 ^is also  i.e. 1 genetic s.d. within each line. Then, assuming both lines have an accumulated inbreeding coefficient of F since the base populations, equating expectations:

since it is assumed the difference is a result of drift. On the right hand side, *cov*(*μ*_*R*_, *μ*_*D*_) = 0, and . Furthermore . Therefore:

to give F = 0.2. Therefore where the observed result for the squared difference would have been 'just as expected' occurs when F = 0.2, which gives 

The Mendelian sampling variance of an offspring within this framework is given by

The F1 offspring have F = 0.0, but F_sire _= F_dam _= 0.2 since they come from within the recipient and donor lines and have accumulated inbreeding, .

For BC1, the offspring has 1/2 chance of receiving two randomly selected recipient alleles with a probability of identity by descent (IBD) of 0.2, and 1/2 chance of receiving one recipient and one donor allele with a probability 0 of being IBD, so the offspring has F = 0.1. It has one parent with F = 0.0 and the other with F = 0.2, to give

For BC2, for a locus unlinked to that being introgressed, the offspring has probability ¾ of receiving two randomly selected recipient line alleles with a probability of identity by descent (IBD) of 0.2, and probability 1/4 of receiving one recipient and one donor line allele with probability 0 being IBD, so the offspring has F = 0.15. It has one parent with F = 0.1 and the other with F = 0.2, to give

This sequence continues for BC3 and BC4 analogously, and the offspring have inbreeding coefficients of 0.175 and 0.1875 respectively, and  and 

## Competing interests

The authors declare that they have no competing interests.

## Authors' contributions

MHY derived and implemented the methods, created and analysed the simulation study, and wrote the paper. Approach for calculating variance within lines derived and wrote by JAW in the appendix. AKS, JAW, and THEM conceived the study, took part in discussions, and provided input to the writing of the paper. All authors have read and approved the paper.
